# Supplementary material for: Should I Stay or Should I Go? Associations between Occupational Factors, Signs of Exhaustion, and the Intention to Change Workplace among Swedish Principals
Source: Int J Environ Res Public Health. 2021 May 18;18(10):5376. doi: 10.3390/ijerph18105376 (PMC8158094; doi:10.3390/ijerph18105376)
Supplement: Supplementary file 1 [file ijerph-18-05376-s001.zip › Supplementary Table S1.pdf]

Supplementary Table S1. Description of the scales in the 32-item Gothenburg Manager Stress Inventory (GMSI)-Mini[a].

Demanding factors	Description	Number of items
Resource deficits	Insufficient possibilities of affecting the allocation of resources to the organisation; lacking resources due to decisions by superiors, politicians, or governmental authorities; not enough resources to cope with peak loads.	3
Organisational control	Severe difficulties in implementing the decisions from superior levels in the organisation; difficulties following how decisions are made in the organisation.	2
Role conflicts	Conflicts between administrative work, organisational development and co-workers; not enough time for organisational development; difficulties in finding the time to discuss daily activities with colleagues	3
Role demands	Demanding responsibilities for (a) performance and quality; (b) personnel; (c) the work environment; and (d) organisational development.	4
Group dynamics	Problems with feelings of safety and mutual trust within the co-worker group; feelings of not knowing what is going on in your co-worker group; Co-workers having trouble accepting the mutual work goals.	3
Buffer-function	Demands of being a buffer between co-workers and higher levels in the organisation; demands of having to explain "bad/negative" decisions that have been made by superiors; superiors expecting you to be understanding and committed to accepting decisions that are bad for you and your organisation.	3
Co-workers	Demands on helping co-workers organise and structure their work; that co-workers have insufficient structure in their work.	2
Container-function	Demands of dealing with co-workers' frustrations that work is psychologically challenging; that burdened co-workers burden you with their problems.	2
**Supportive factors**		
Supportive management	Trusting that superiors, when needed, will help me to solve work environment problems for my co-workers; experiencing that superiors express a genuine interest for what I do and the problems I have as a leader.	2
Cooperating co-workers	Feeling that co-workers want to take responsibility for their work; feeling that co-workers have valuable knowledge that makes my work easier.	2
Supportive manager colleagues	When needed, I have access to proper support from my fellow school leader colleagues; I have true possibilities to reflect and discuss organisational issues with my fellow school leader colleagues.	2
Supportive private life	My leisure time interests facilitate relaxation from work and associated problems; my leisure time really provides me with opportunities to rest and relax from work.	2
Supportive organisational structures	My authority in my work is clear and defined; my area of responsibility and tasks as a leader are clear and defined.	2

[a]This information has previously been presented in Persson et al [15].
