# Supplementary material for: Should I Stay or Should I Go? Associations between Occupational Factors, Signs of Exhaustion, and the Intention to Change Workplace among Swedish Principals
Source: Int J Environ Res Public Health. 2021 May 18;18(10):5376. doi: 10.3390/ijerph18105376 (PMC8158094; doi:10.3390/ijerph18105376)
Supplement: Supplementary file 1 [file ijerph-18-05376-s001.zip › Supplementary Table S2.pdf]

Supplementary Table S2. Changes of reported exposures (2018 *vs.* 2019) in group 7 (Leave/ Change/ Stay) and group 8 (Leave/ Change/ Leave) (Figure 1); comparison of exposures between group 7 and group 8 (2018 and 2019). Results in bold face are statistically significant.

		Group 7 (Leave/ Change/ Stay) N=21			Group 8 (Leave/ Change/ Leave) N=19			Difference between Group 7 and Group 8 p-value[A]	
Independent factors	Categories/Scale	**2018**	**2019**	2018 *vs.* 2019; p-value[B]	**2018**	**2019**	2018 *vs.* 2019; p-value[B]	**2018**	**2019**
Gender; N (%)	*Female*	15 (71)			10 (53)			0.33	
	Male	6 (29)			9 (47)				
Age (2018); mean (SD)	*years*	47 (5)			47 (7)			0.54	
School owner; N (%)	*Municipality*	18 (86)	21 (100)	0.25	18 (95)	16 (84)	0.50	0.61	0.10
	Other organisation	3 (14)	0		1 (5)	3 (16)			
Job title; N (%)	*Principle*	16 (76)	17 (81)	1.0	13 (68)	12 (63)	1.0	0.73	0.70
	Assistant principle	5 (24)	4 (19)		6 (32)	5 (26)			
	Other title[C]	-	0		-	2 (11)			
Number of co-workers; mean (SD)		30 (11)	32 (13)	0.49	34 (15)	29 (11)	0.54	0.50	0.56
Staff access/availability; N (%)	*Full staff*	13 (62)	15 (71)	0.73	7 (37)	11 (58)	0.22	0.20	0.51
	Very or somewhat understaffed	8 (38)	6 (29)		12 (63)	8 (42)			
Number of students; mean (SD)		218 (88)	266 (171)	0.73	252 (106)	253 (150)	0.86	0.25	0.89
Overtime; N (%)	*Once a week or less often*	4 (19)	3 (14)	0.66	2 (11)	2 (11)	0.32	0.66	1.00
	Every day or a few days/week	17 (81)	18 (86)		17 (89)	15 (79)			
	No agreed working hours[D]	0	0		0	2 (11)			
Physical working environment; N (%)	*Adequate, good or very good*	21 (100)	17 (81)	0.12	8 (42)	13 (68)	0.23	**<0.001**	0.47
	Poor or very poor	0	4 (19)		11 (58)	6 (32)			

**Demanding organisational and social work environment; mean (SD)**									
Resource deficits	Score 1-5	3.5 (0.8)	3.5 (1.2)	0.67	3.8 (0.8)	3.3 (1.1)	**0.04**	0.22	0.44
Organisational Control	Score 1-5	2.5 (1.0)	2.7 (1.0)	0.32	3.1 (0.8)	2.9 (0.8)	0.39	**0.04**	0.50
Role conflicts	Score 1-5	3.7 (1.0)	3.6 (0.8)	0.65	4.3 (0.6)	3.8 (0.8)	**0.02**	0.05	0.49
Role demands	Score 1-5	3.0 (0.9)	2.8 (0.7)	0.21	3.4 (0.6)	3.2 (0.6)	0.16	0.26	0.08
Group dynamics	Score 1-5	2.2 (0.8)	2.3 (0.7)	0.61	2.9 (0.9)	2.6 (0.5)	0.21	**0.02**	0.07
Buffer-function	Score 1-5	2.8)0.7)	2.6 (0.8)	0.48	3.6 (0.8)	3.5 (0.9)	0.24	**0.001**	**0.01**
Co-workers	Score 1-5	2.7 (0.6)	2.9 (0.6)	0.11	3.2 (0.9)	3.3 (0.6)	0.75	**0.05**	0.08
Container-function	Score 1-5	3.1 (0.8)	3.2 (0.8)	0.71	3.9 (0.6)	3.7 (0.8)	0.28	**0.003**	0.09
**Supportive organisational and social work environment; mean (SD)**									
Supportive management	Score 1-5	2.9 (1.0)	3.3 (1.0)	0.07	2.7 (0.9)	2.7 (1.1)	0.87	0.67	0.06
Cooperating with co-workers	Score 1-5	4.5 (0.5)	4.2 (0.6)	0.10	4.1 (0.5)	4.0 (0.7)	0.57	**0.01**	0.38
Supportive colleagues	Score 1-5	3.7 (1.0)	3.7 (0.9)	0.79	2.9 (1.4)	3.5 (1.0)	**0.04**	0.05	0.50
Supportive private life	Score 1-5	3.7 (0.9)	4.2 (0.8)	**0.01**	3.5 (1.4)	3.8 (1.2)	0.12	0.92	0.35
Supportive organisational structures	Score 1-5	3.3 (0.9)	3.5 (1.0)	0.20	3.3 (0.7)	2.9 (1.0)	**0.04**	0.69	0.09
**Stressful external expectations; mean (SD)**									
The National Agency of Education	Score 1-7	3.0 (1.9)	3.4 (1.7)	0.41	4.0 (1.6)	3.4 (1.9)	0.21	0.12	0.89
The Swedish school-inspectorate	Score 1-7	4.3 (2.1)	5.2 (1.6)	0.07	4.6 (1.7)	4.5 (2.0)	0.64	0.73	0.27
School owner	Score 1-7	3.8 (1.8)	3.8 (1.4)	0.93	4.7 (1.6)	4.7 (1.6)	0.97	0.17	**0.03**
Super Intendent	Score 1-7	3.0 (1.6)	3.4 (1.6)	0.29	4.1 (1.6)	3.9 (1.9)	0.70	**0.04**	0.32
Immediate supervisor	Score 1-7	3.1 (1.5)	3.0 (1.4)	0.81	3.9 (1.5)	4.3 (1.6)	0.34	0.14	**0.01**
Co-workers	Score 1-7	3.9 (1.5)	4.1 (1.2)	0.56	4.9 (1.5)	4.0 (1.8)	0.08	0.05	0.94
Parents	Score 1-7	4.7 (1.8)	4.6 (1.4)	0.67	5.0 (1.7)	4.7 (1.8)	0.50	0.71	0.57
Students	Score 1-7	2.8 (1.7)	2.8 (1.6)	0.89	3.5 (1.8)	2.6 (1.4)	**0.02**	0.23	0.67
**Karolinska Exhaustion Disorder Scale (KEDS)**									
Mean score (SD)	Score 0-54	14 (8.5)	13 (9)	0.94	17 (7)	17 (10)	0.69	0.08	0.39
Possible exhaustion disorder; N (%)	Score ≥ 19 points	6 (29)	3 (14)	0.25	9 (47)	9 (47)	1.0	0.33	**0.04**

[A] The Mann Whitney U-test was used for continuous variables and Fisher's exact test was used for dichotomous variables.

[B] The Wilcoxon Signed Rank Test was used for continuous variables and McNemar's test for dichotomous variables.

[C] Two individuals with ”other title” in 2019 were excluded from the statistical analysis.
[D] Participants with “No agreed working hours” were excluded from the statistical analysis.
